# Phylogenetic analysis of *Dianthus* using ITS and RAPD markers reveals insights for carnation breeding

**DOI:** 10.1186/s40529-025-00475-x

**Published:** 2025-12-03

**Authors:** Carlo Mascariola Cabillo, Yuan-Hua Tuan, Yen-Ming Chen, Yu-Ting Huang, Chia-Wen Li, I-Chun Pan

**Affiliations:** 1 Department of Horticulture, National Chung Hsing University, Taichung, Taiwan; 2 Department Agricultural Sciences, Southern Leyte State University-Bontoc Campus, Southern Leyte, Bontoc, 6604 Philippines; 3 Department of Life Science, Fu Jen Catholic University, New Taipei City, Taiwan; 4 Advanced Plant and Food Crop Biotechnology Center, National Chung Hsing University, Taichung, 402 Taiwan

**Keywords:** *Dianthus*, Molecular markers, ITS, RAPD

## Abstract

**Background:**

Carnation and *Dianthus* (*Dianthus* spp) are globally cultivated as a cut flower, yet high summer temperatures in Taiwan significantly reduce the yield and quality of commercial cultivars. To enhance stress tolerance traits such as heat resistance and disease resistance, interspecific and intergeneric hybridization with native species is a viable approach. Since the success of hybridization is influenced by genetic distance, this study aimed to clarify the phylogenetic relationships among native species, commercial cultivars, and interspecific hybrids using internal transcribed spacer (ITS) and random amplified polymorphic DNA (RAPD) markers.

**Results:**

Phylogenetic analysis based on RAPD markers effectively differentiated among native Taiwanese species, commercial varieties, interspecific hybrids, and outgroup taxa. ITS markers, on the other hand, were more informative for identifying parent-offspring relationships. Notably, *Dianthus superbus* var. *longicalycinus* from Taiwan and Japan, despite sharing the same scientific name, exhibited clear distinctions in both morphological traits and molecular profiles.

**Conclusions:**

The combined use of RAPD and ITS markers provides complementary insights into the genetic relationships within the *Dianthus* genus. These findings not only support the strategic use of molecular markers in breeding programs but also highlight the need to reassess taxonomic classifications among morphologically similar varieties. This study provides genetics, breeding tools, and germplasm information for future *Dianthus* breeding.

## Introduction

The floral industry plays a significant role in the economies of many countries and meets the aesthetic needs of local residents. Developing countries widely adopt cut flower production as a successful economic growth strategy and as a means of creating jobs and generating foreign exchange income (Linares-Gabriel et al. 2020; Patel-Campillo 2011). Carnations, scientifically known as *Dianthus caryophyllus*, are important cut flowers worldwide, with their quality and yield significantly influenced by the growing environment (Sharma et al. 2020). Carnations are also one of the main cut flowers cultivated and produced in Taiwan, but some commercial varieties exhibit poor growth under the high temperatures of Taiwan’s summer.

The geographical distribution of a species greatly influences its functional traits, environmental adaptation, relative performance, and competition among species as a survival mechanism (Bruno et al. 2000). Genetic trait variability increases as a result of climatic conditions compared to related species or the same species (Grime and Pierce 2012). Several native *Dianthus* species were found in Taiwan. *D*. *pygmaeus* and *D*. *seisuimontanus* grow at altitudes above 2000 m, while *D*. *palinensis*, unknown *Dianthus* spp. (Mt. Da-du), and unknown *Dianthus* spp. (Miaoli County) are found in low-altitude areas (Lee 2004). *Dianthus superbus* (Taiwan) L. var. *longicalycinus* was found on Matsu Island, which is a relatively isolated and closed ecosystem (Bao-Yi 2007). Among these, *Dianthus palinensis* can adapt to hot and humid climates and exhibits disease resistance traits. *Dianthus palinensis* grows in the northern part of Taiwan and can flower year-round on flat ground (Lee 2004). By interspecies hybridization of Taiwan endemic species, valuable traits such as the ability to over-summer, long vase life, and disease resistance could be introduced to commercial cultivars.

Interspecific or intergeneric crosses are applied to the breeding of ornamental plants, and cross-compatibility is affected by genetic distance (Huang et al. 2002). The traditional classification of *Dianthus* species was based on morphological characteristics among species to determine their genetic relative distance, but this leads to uncertain identification. Genetic markers can be used to study hereditary patterns, genetic recombination, gene mapping relationships, and species identification more precisely (Hayward et al. 2015). Molecular markers have been used in several scientific investigations to pinpoint the origin and traits that distinguish a particular organism. Polymerase chain reaction (PCR)-based molecular markers have been used as a tool in breeding programs (Mukherjee et al. 2015). Moreover, DNA barcoding, known as DNA taxonomy, is based on PCR-amplified DNA sequences that serve as a baseline to identify a species or its taxon (Tautz et al. 2003). Many researchers utilize molecular markers because they are relatively stable and can reduce or avoid classification confusion caused by environmental and phenotypic changes (Agarwal et al. 2008). This technique accelerates the verification of a species’ origin and genetic traits based on its whole genome sequence.

Molecular phylogenetic analysis has proven essential for resolving species ambiguities within the Caryophyllaceae family, which are often difficult to identify solely through morphological characteristics (Fior et al. 2006). The internal transcribed spacer (ITS) region of nuclear ribosomal DNA (nrDNA), situated between the 18 S and 26 S subunits, is commonly used for both phylogenetic studies and species identification across related genera (Baldwin et al. 1995; Volkov et al. 2007). Molecular-based taxa were established by combining a chloroplast *matK* gene and ITS molecular marker, which successfully separated three subfamilies of Caryophyllaceae including Alsinoideae, Caryophylloideae, and Paronychioideae (Fior et al. 2006). Recently, ITS have been used for the species identification of Korean Caryophyllaceae. The use of ITS achieved over 73% accuracy in species identification across the family and specifically provided 60% species resolution in *Dianthus* (Jin et al. 2023).

Random amplified polymorphic DNA (RAPD) have also been widely applied for DNA fingerprinting and population genetic studies (Hasan et al. 2021; Madhumati 2014). For example, Wu et al. (2003) demonstrated RAPD’s ability to distinguished between wild species and cultivars of *Dianthus*, highlighting its potential for germplasm management and breeding strategies in this species (Wu 2003). RAPD markers were employed to assess genetic divergence in two *Dianthus carthusianorum* populations from polluted and unpolluted soil. The study confirmed that the distinct genetic variations observed between the populations corresponded to their morphological diversity, highlighting the effectiveness of RAPD in detecting such differences (Wójcik et al. 2013). Additionally, RAPD has proven to be an efficient tool, capable of detecting various molecular markers in hybrid cultivars of *Dianthus* (Jayed et al. 2022).

Molecular markers have been researched for breeding purposes for many years. However, the applicability of different molecular markers may vary depending on the crop species being studied. To explore the applicability of different molecular markers in *Dianthus* spp., 14 species or cultivars were selected. In this study, six native Taiwanese species, including two unnamed *Dianthus* spp., one native Japanese species, three commercial hybrids of Chrysanthemum in Taiwan, four interspecific hybrid species, and two outgroup plants were collected. ITS and RAPD were conducted as molecular markers to distinguish, identify and analyze genetic diversity and to establish a genetic database for future breeding programs.

## Results

### Morphological characteristics

**Fig. 1:**
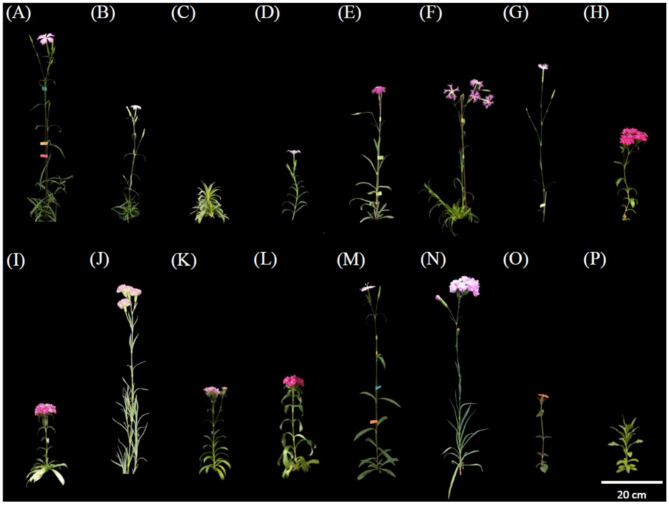
Features of 14 species of genus *Dianthus* and 2 outgroups. Taiwan endemic species include (**A**) *D*. *palinensis*, (**B**) *D*. *pygmaeus*, (**C**) *D*. *seisuimontanus*, (**D**) *D*. *superbus* (Taiwan) L. var. longicalycinus, (**E**) *D*. *superbus* (Japan) L. var. longicalycinus, (**F**) unknown *D*. *spp*. (Mt. Da-du), and (**G**) unknown *D*. *spp*. (Miaoli County), commercial cultivars include (**H**) *D*. *japonicus* ‘Red Plum’, (**I**) *D*. *barbatus* ‘Lord Breanthus’, and (**J**) *D*. *caryophyllus* ‘Festival’, hybrid cultivars include (**K**) *D*. *japonicus* ‘Red Plum’ × *D*. *palinensis*, (**L**) *D*. *barbatus* × *D*. *japonicus* ‘Red Plum’, (**M**) *D*. *japonicus* ‘Red Plum’ × *D*. *caryophyllus* ‘Festival’, and (**N**) *D*. *caryophyllus* ‘Festival’ × *D*. *palinensis*, and outgroups include (**O**) *Lychnis fulgens* (**P**) *Saponaria officinalis*

The morphological characteristics of *Dianthus* species were shown in Table 1. Among the seven native species, *D. palinensis*, *D*. *superbus* (Japan) L. var. *longicalycinus*, unknown *Dianthus spp.* (Mt. Da-du), and unknown *Dianthus spp.* (Miaoli County) had relatively taller plants (Fig. 1A–F). *D*. *caryophyllus* ‘Festival’ was the tallest among the three commercial cultivars (Fig. 1H–J), and it’s two hybrid offspring, *D*. *japonicus* ‘Red Plum’ × *D*. *caryophyllus* ‘Festival’ and *D*. *caryophyllus* ‘Festival’ × *D*. *palinensis*, maintain the plant height characteristics (Fig. 1M, N).

**Table 1:** Morphological characteristics of *Dianthus* species

taxon	cultivar/line	Height (cm)	Leaf			Inflorescence	
			Shape	Length (cm)	Width (mm)	Type	Diameter (cm)
**Native Taiwanese and Japanese Species**							
	*Dianthus palinensis*	56–60	Lanceolate	5–10	8–9	cyme	4–4.5
	*Dianthus pygmaeus*	20–24	Lanceolate	4–5	2–3	cyme	2–2.5
	*Dianthus seisuimontanus*	8–12	Linear	7–8	7–7.5	cyme	2–2.5
	*Dianthus superbus* (Taiwan) L. var. *longicalycinus*	18–23	Lanceolate	4–5	8–10	cyme	4–4.5
	*Dianthus superbus* (Japan) L. var. *longicalycinus*	17–29	Linear-lanceolate	9	5.4–5.8	cyme	4–4.5
	Unknown *Dianthus* spp. (Mt. Da-du)	45–60	Lanceolate	6–7	1.2–1.4	cyme	4–4.5
	Unknown *Dianthus* spp. (Miaoli County)	48–60	Linear	12–13	3–4	cyme	3.5–4
**Commercial Cultivars**							
	*Dianthus japonicus* ‘Red Plum’	40–42	Lanceolate	8–9	8–9	cyme	2
	*Dianthus barbatus* ‘Lord Breanthus’	27–29	Lanceolate	8–9	12–13	umbel	3
	*Dianthus caryophyllus* ‘Festival’	42–44	Linear	7.5-9	6.5-8	cyme	5
**Interspecific Hybrid Species**							
	*D. japonicus* ‘Red Plum’ *× D. palinensis*	42–44	Lanceolate	6–7	5–6	cyme	3
	*D. barbatus × D. japonicus* ‘Red Plum’	33–35	Lanceolate	6	18–19	umbel	3
	*D. japonicus* ‘Red Plum’ *× D. caryophyllus* ‘Festival’	56–58	Lanceolate	9	6	cyme	3
	*D. caryophyllus* ‘Festival’ *× D. palinensis*	54–56	Lanceolate	9	4–5	cyme	6

**Fig. 2:**
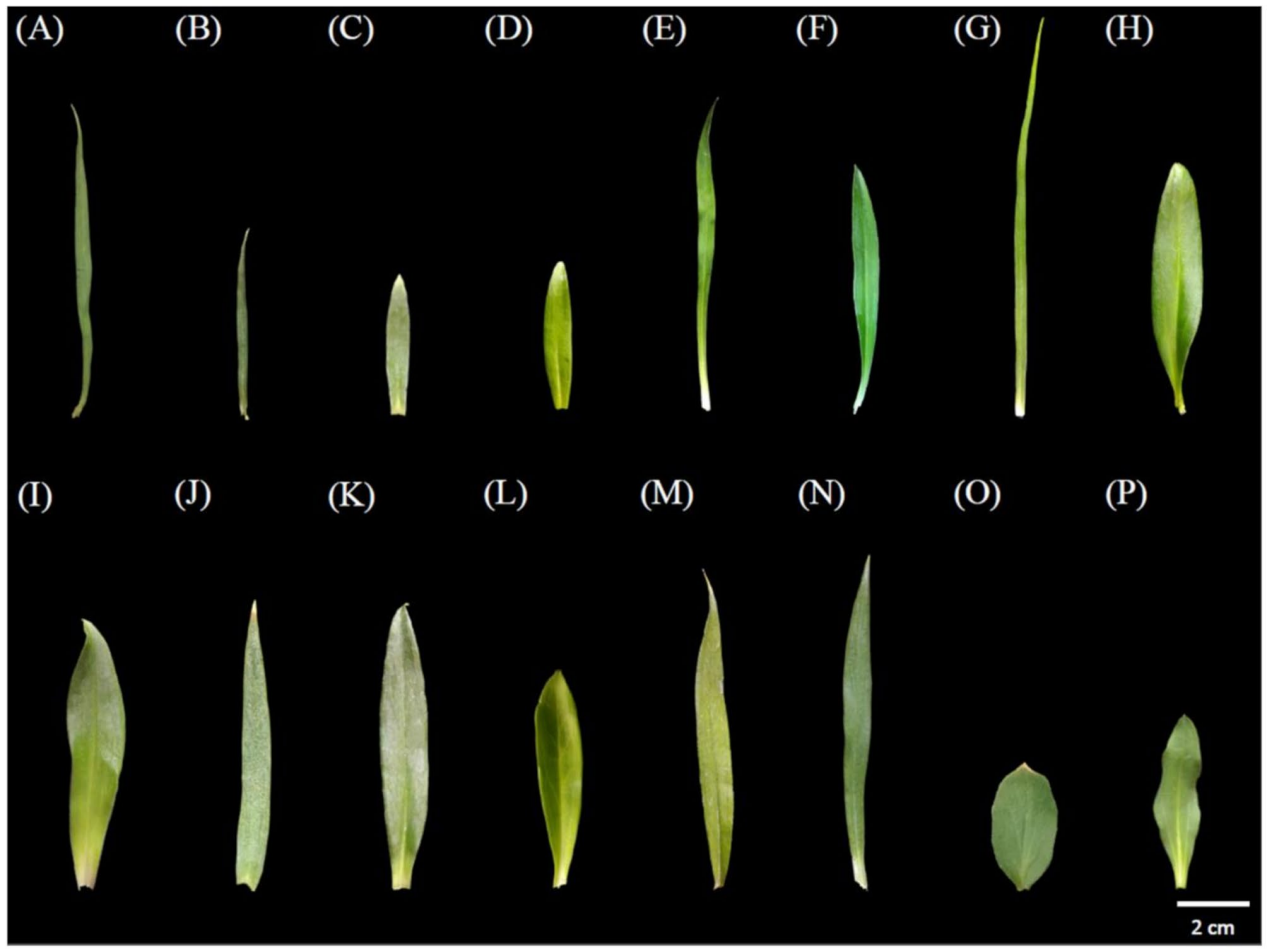
Leaf features of 14 species of genus *Dianthus* and 2 outgroup. Taiwan endemic species include (**A**) *D*. *palinensis*, (**B**) *D*. *pygmaeus*, (**C**) *D*. *seisuimontanus*, (**D**) *D*. *superbus* (Taiwan) L. var. longicaycinus, (**E**) *D*. *superbus* (Japan) L. var. longicaycinus, (**F**) unknown *D*. *spp*. (Mt. Da-du), and (**G**) unknown *D*. *spp*. (Miaoli County), commercial cultivars include (**H**) *D*. *japonicus* ‘Red Plum’, (**I**) *D*. *barbatus* ‘Lord Breanthus’, and (**J**) *D*. *caryophyllus* ‘Festival’, hybrid cultivars include (**K**) *D*. *japonicus* ‘Red Plum’ × *D*. *palinensis*, (**L**) *D*. *barbatus* × *D*. *japonicus* ‘Red Plum’, (**M**) *D*. *japonicus* ‘Red Plum’ × *D*. *caryophyllus* ‘Festival’, and (**N**) *D*. *caryophyllus* ‘Festival’ × *D*. *palinensis*, and outgroups include (**O**) *Lychnis fulgens* (**P**) *Saponaria officinalis*

*Dianthus* plants exceeding 40 cm in height, such as plants *D*. *palinensis*, *D*. *superbus* (Japan) L. var. *longicalycinus*, unknown *D*. spp. (Mt. Da-du), unknown *D*. spp. (Miaoli County), *D*. *caryophyllus* ‘Festival’, *D*. *japonicus* ‘Red Plum’ × *D. caryophyllus* ‘Festival’, and *D*. *caryophyllus* ‘Festival’ × *D. palinensis* (Fig. 1A, E–G, J, M, N), have leaves longer than 6 cm (Fig. 2A, E–G, J, M, N). However, shorter *Dianthus* plants do not necessarily have shorter leaves. For instance, *D*. *barbatus* ‘Lord Breanthus’, and *D*. *japonicus* ‘Red Plum’ × *D. palinensis*, which are approximately 20 cm tall (Fig. 1I, K), have leaves longer than 7 cm (Fig. 2I, K).

**Fig. 3:**
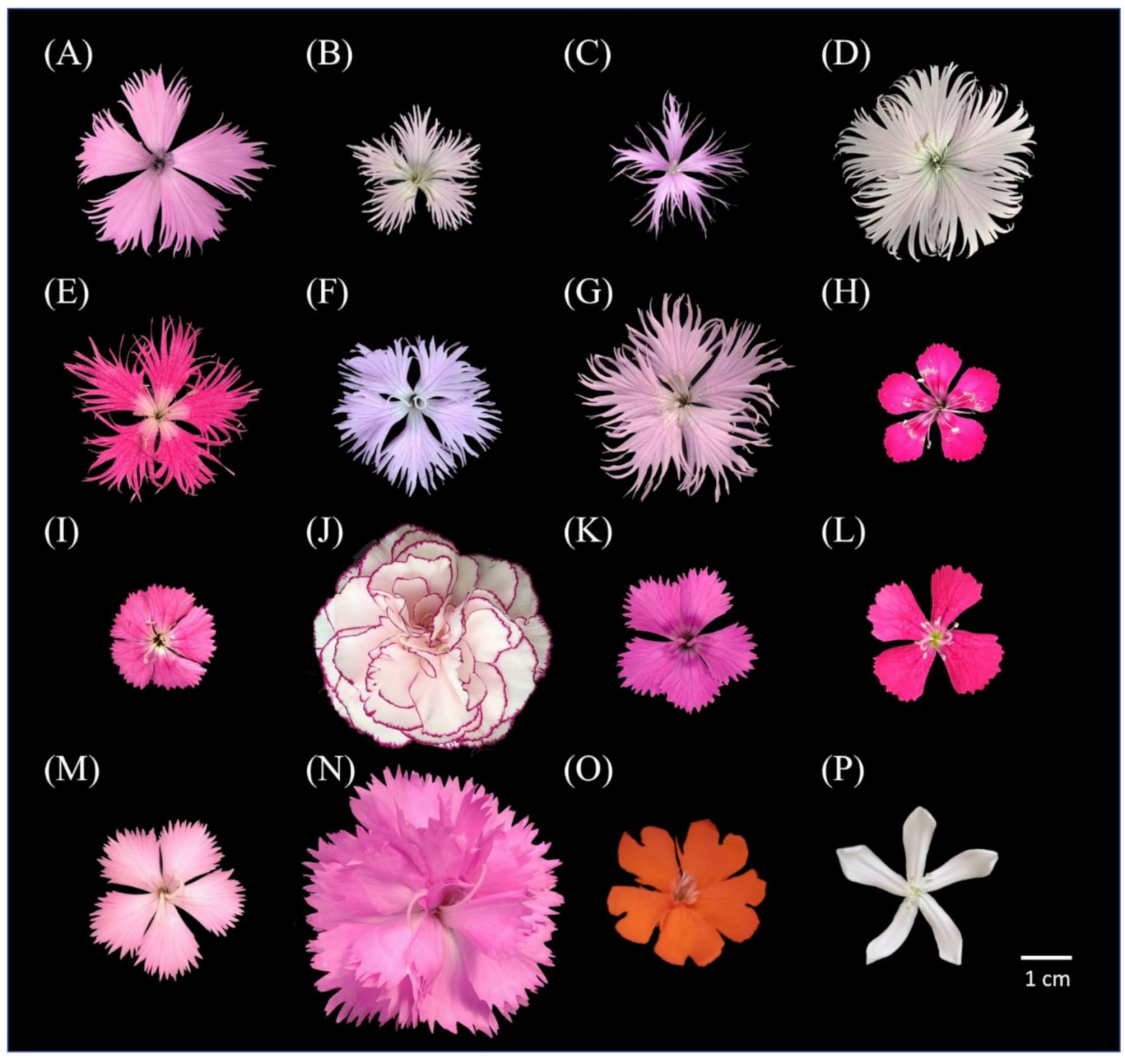
Floral features of 14 species of genus *Dianthus* and 2 outgroup. Taiwan endemic species include (**A**) *D*. *palinensis*, (**B**) *D*. *pygmaeus*, (**C**) *D*. *seisuimontanus*, (**D**) *D*. *superbus* (Taiwan) L. var. longicaycinus, (**E**) *D*. *superbus* (Japan) L. var. *longicaycinus*, (**F**) unknown *D*. *spp*. (Mt. Da-du), and (**G**) unknown *D*. *spp*. (Miaoli County), commercial cultivars include (**H**) *D*. *japonicus* ‘Red Plum’, (**I**) *D*. *barbatus* ‘Lord Breanthus’, and (**J**) *D*. *caryophyllus* ‘Festival’, hybrid cultivars include (**K**) *D*. *japonicus* ‘Red Plum’ × *D*. *palinensis*, (**L**) *D*. *barbatus* × *D*. *japonicus* ‘Red Plum’, (**M**) *D*. *japonicus* ‘Red Plum’ × *D*. *caryophyllus* ‘Festival’, and (**N**) *D*. *caryophyllus* ‘Festival’ × *D*. *palinensis*, and outgroups include (**O**) *Lychnis fulgens* (**P**) *Saponaria officinalis*

The depth of the indentation on the petal margin is an important taxonomic characteristic of *Dianthus* flowers. The petals of all the native species have deep petal margin indentations (Fig. 3A–G). Using the native species *D*. *palinensis* (Fig. 3A) as a parent, the offspring *D*. *japonicus* ‘Red Plum’ × *D. palinensis* (Fig. 3K) and *D*. *caryophyllus* ‘Festival’ × *D. palinensis* (Fig. 3N) also exhibit noticeable petal margin indentations. The commercial variety *D*. *caryophyllus* ‘Festival’ (Fig. 3J) has double petals, and when *D*. *caryophyllus* ‘Festival’ is used as either the mother or father, the offspring have single petals (*D*. *caryophyllus* ‘Festival’ × *D. palinensis*, Fig. 3N) and double petals (*D*. *japonicus* ‘Red Plum’ × *D. caryophyllus* ‘Festival’, Fig. 3M), respectively.

### *Dianthus* ITS phylogenetic analysis

**Fig. 4:**
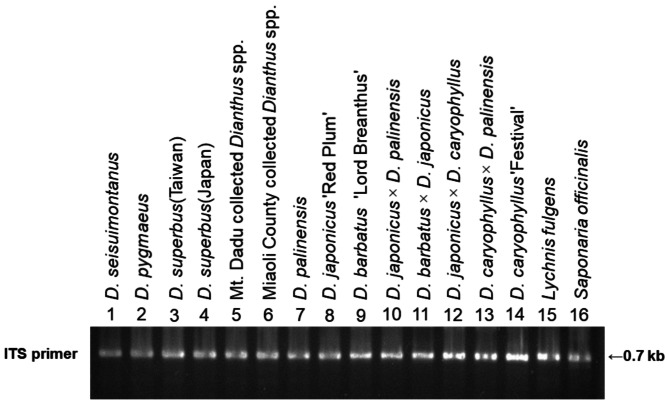
Amplification profile of 16 accessions with the ITS primer

**Fig. 5:**
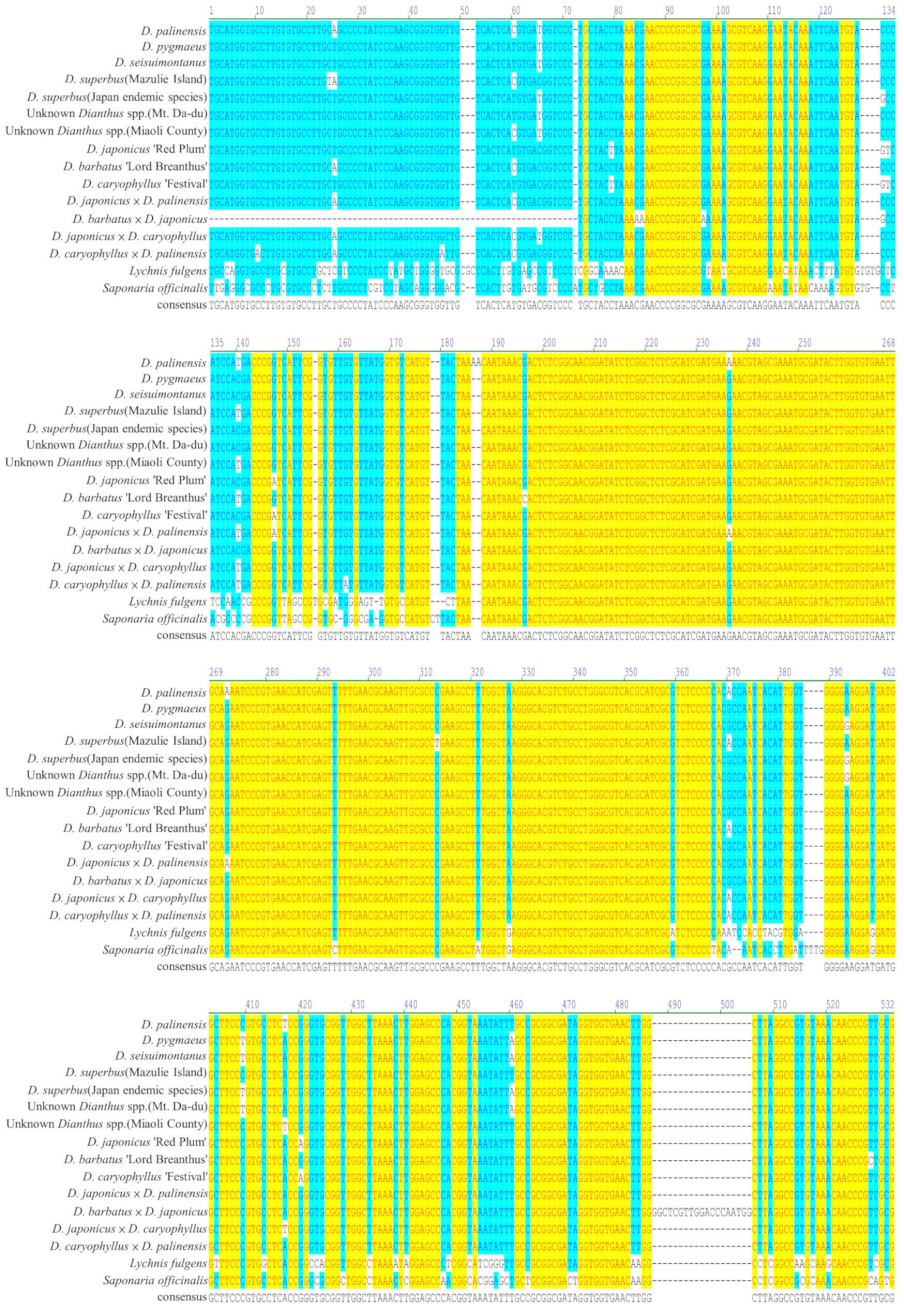
The alignment sequence of 14 *Dianthus* and 2 outgroups in ITS, ITS1-ITS2

Using PCR, we obtained the ITS fragments of 16 *Dianthus* species and 2 outgroup plants (Fig. 4). It was found that the two outgroup plants, *Lychnis fulgens* and *Saponaria officinalis*, have significantly larger DNA differences compared to the 16 *Dianthus* species. *D*. *barbatus* × *D*. *japonicus* has a noticeable deletion in the sequence at the front end, but there is an additional 19 bp DNA fragment at the rear end. Most *Dianthus* species exhibit 1 bp sequence differences at various sites (Fig. 5).

**Fig. 6:**
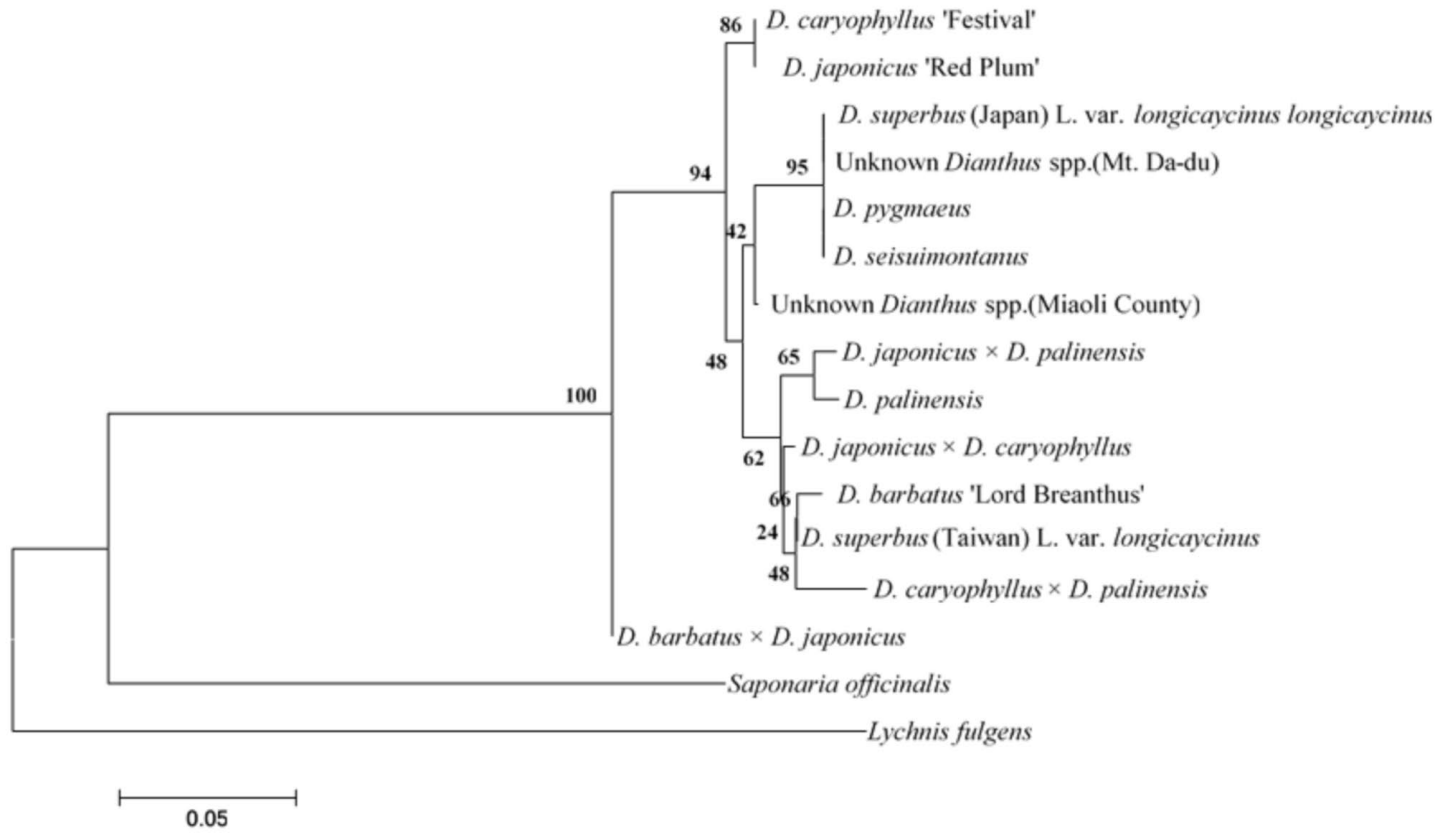
Phylogenetic analysis based on ITS sequence. The numbers indicate the percentage of bootstrap value for each node. X- axis of the phylogenetic tree represents the distance or dissimilarity between clusters. Y- axis represents the objects and clusters

Phylogenetic analysis shows that, except for the outgroup plants, *D*. *barbatus* × *D*. *japonicus* is clearly separated from the other 13 *Dianthus* species. Among the remaining 13 *Dianthus* species, the two commercial varieties, *D*. *japonicus* ‘Red Plum’ and *D*. *caryophyllus* ‘Festival’, form their own distinct group. All seven native Taiwanese species, the commercial variety *D*. *barbatus* ‘Lord Breanthus’, and the three hybrid varieties are grouped together. The two identically named species, *D*. *superbus* (Taiwan) L. var. *longicalycinus* and *D*. *superbus* (Japan) L. var. *longicalycinus*, are also clearly distinguished from each other (Fig. 6).

### RAPD analysis of *Dianthus*

**Fig. 7:**
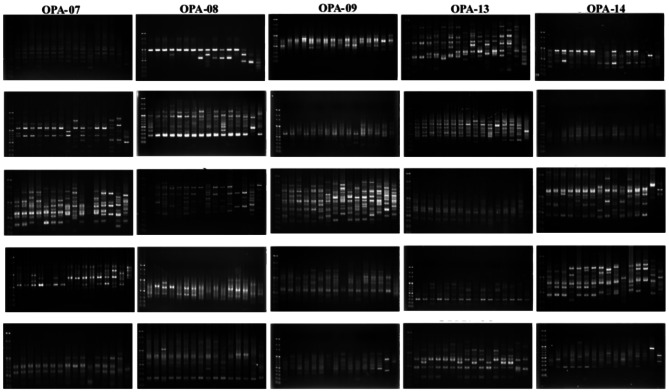
RAPD amplification profile of 16 accessions with 25 RAPD primers. Lane identifications of each result from left to right are: 1. Marker, 2. *D*. *palinensis*, 3. *D*. *pygmaeus*, 4. *D*. *seisuimontanus*, 5. *D*. *superbus* (Taiwan) L. var. longicaycinus, 6. *D*. *superbus* (Japan) L. var. longicaycinus, 7. unknown *D*. *spp*. (Mt. Da-du), 8. unknown *D*. *spp*. (Miaoli County), 9. *D*. *japonicus* ‘Red Plum’, 10. *D*. *barbatus* ‘Lord Breanthus’, 11. *D*. *caryophyllus* ‘Festival’, 12. *D*. *japonicus* ‘Red Plum’ × *D*. *palinensis*, 13. *D*. *barbatus* × *D*. *japonicus* ‘Red Plum’, 14. *D*. *japonicus* ‘Red Plum’ × *D*. *caryophyllus* ‘Festival’, 15. *D*. *caryophyllus* ‘Festival’ × *D*. *palinensis*, 16. *Lychnis fulgens* and 17. *Saponaria officinalis*

**Fig. 8:**
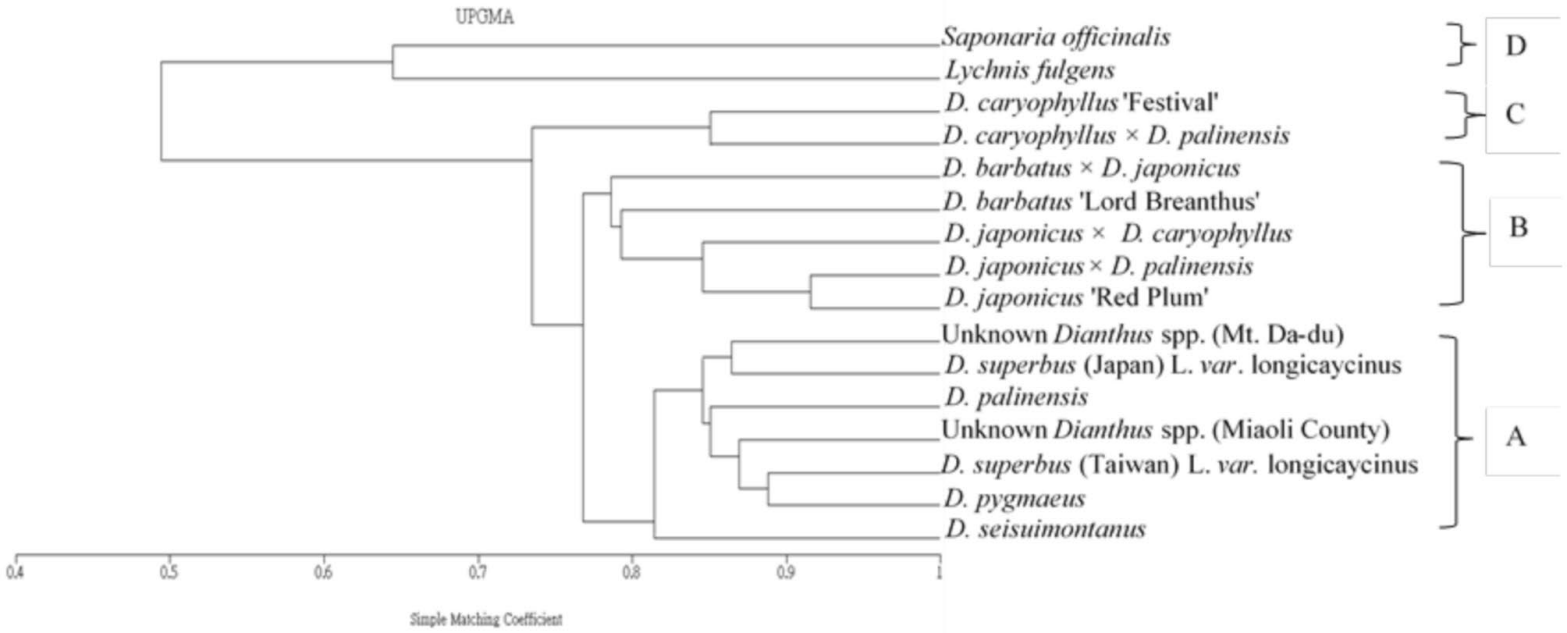
Phylogenetic analysis of RAPD. The phylogenetic tree was based on products of Random Amplified Polymorphic DNA (RAPD). X- axis of the phylogenetic tree represents the distance or dissimilarity between clusters. Y- axis represents the objects and cluster

Polymorphism was observed in 16 *Dianthus* species and two outgroups by using 25 sets of RAPD primers for PCR (Fig. 7). Phylogenetic analysis clearly distinguishes the 2 outgroups from the *Dianthus* species (Fig. 8D). The 16 *Dianthus* species can be divided into three groups: the seven native Taiwanese species form one group (Fig. 8A); commercial varieties or hybrids containing *D*. *japonicus* ‘Red Plum’ or *D*. *barbatus* ‘Lord Breanthus’ lineage form another group (Fig. 8B); and those containing *D*. *caryophyllus* ‘Festival’ lineage constitute a separate group (Fig. 8C).

## Discussion

Plant morphology is one of the classical methods for plant classification, with the genus *Dianthus* typically classified based on plant size, leaf shape, and calyx shape. However, leaf length and width in *Dianthus* can change due to environmental factors, leading to confusion in identification and classification (Lee 2004). Another morphological basis for *Dianthus* classification is the degree of petal margin indentation. The hybrid variety *D*. *japonicus* × *D*. *palinensis* exhibits petal margin indentation that is intermediate between its parents, *D*. *japonicus* ‘Red Plum’ and *D*. *palinensis* (Fig. 3A, H, K). Although *D*. *palinensis* is the only native *Dianthus* with shallow petal margin indentations, while other native *Dianthus* species have deeper indentations, making it difficult to distinguish between them (Fig. 3A–G). Plant morphology can indeed serve as a basis for classification to a certain extent, but accurately conducting phylogenetic identification remains challenging.

The Taiwanese native species *D*. *superbus* (Taiwan) L. var. *longicalycinus* and the Japanese native species *D*. *superbus* (Japan) L. var. *longicalycinus* share the same scientific name *D*. *superbus* L. var. *longicalycinus* (Fig. 1D, E), but their plant and flower morphologies are very different (Fig. 3D, E). *D*. *superbus* (Taiwan) L. var. *longicalycinus* has lanceolate leaves that are shorter, measuring only 4–5 cm (Fig. 2D), whereas *D*. *superbus* (Japan) L. var. *longicalycinus* has linear-lanceolate leaves that are about 9 cm long, showing significant differences in leaf morphology. RAPD analysis reveals that the species most closely related to *D*. *superbus* (Taiwan) L. var. *longicalycinus* is *Dianthus pygmaeus*, while the species most closely related to *D*. *superbus* (Japan) L. var. *longicalycinus* is the unknown *Dianthus* spp. (Mt. Da-du). The ITS molecular marker results place *D*. *superbus* (Taiwan) L. var. *longicalycinus* and *D*. *superbus* (Japan) L. var. *longicalycinus* in two different groups, indicating that despite sharing a common scientific name, the Taiwanese and Japanese native species should be considered distinct species.

The results of RAPD indicate that all native varieties are classified into the same group (Fig. 7). However, phylogenetic identification using ITS reveals that the native *Dianthus* species *D*. *pygmaeus*, *D*. *seisuimontanus*, *D*. *superbus* (Japan), unknown *Dianthus* spp. (Mt. Da-du), and unknown *Dianthus* spp. (Miaoli County) are closely related. In contrast, two native species, *D*. *palinensis* and *D*. *superbus* (Taiwan) L. var. *longicalycinus*, along with the commercial variety *D*. *barbatus* ‘Lord Breanthus’, which have better heat tolerance under high-temperature cultivation in Taiwan (Data not show), are separately classified into another group (Fig. 6). This result suggests that ITS might be more suitable than RAPD as a screening tool for breeding heat-tolerant *Dianthus* lines.

The results of phylogenetic analysis using ITS indicate that the hybrid offspring *D*. *japonicus* × *D*. *palinensis* and *D*. *japonicus* × *D*. *caryophyllus* are genetically distant from their maternal parent, *D*. *japonicus* ‘Red Plum’. A similar situation is observed with the maternal parent *D*. *barbatus* ‘Lord Breanthus’ and its hybrids. However, hybrids with *D*. *palinensis* as the paternal parent, *D*. *japonicus* × *D*. *palinensis*, show a close genetic relationship, whereas *D*. *caryophyllus* × *D*. *palinensis* is more distantly related (Fig. 6). In contrast, RAPD analysis groups all hybrid offspring with their maternal parents (Fig. 8B, C). Meanwhile, *D*. *caryophyllus* ‘Festival’ × *D*. *palinensis* is closely related to the maternal parent *D*. *caryophyllus* ‘Festival’ but more distantly related to the paternal parent *D*. *palinensis*. This suggests that RAPD might be more effective for identifying the parental origins of hybrid *Dianthus*, particularly for identifying maternal parents.

## Materials and methods

### Plant materials

The test materials were provided by Dr. Yen-Ming Chen’s greenhouse at the Horticultural Experiment Station of National Chung Hsing University (Wufeng District, Taichung City) (24°07′26.7″ N, 120°40′30.2″ E). Experimental materials include six native Taiwanese species, one native Japanese species, three commercial cultivars, and four interspecific hybrid species. Two outgroups *Lychnis fulgens* and *Saponaria officinalis* were also collected. *Lychnis fulgens* is a perennial herb belonging to the Caryophyllaceae family according to the USDA plant database. The plant is growing in the understory of low mountains. The entire plant is softly hairy, with ovate-lanceolate leaves shaped. The petals are deep red, and the flowering period is from June to July (Wu and Raven 1996). *Saponaria officinalis*, a member of the Caryophyllaceae family, stands out with its waxy ovate-lanceolate leaves covered in fine hairs. The flowers of this plant exhibit a spectrum of colors, ranging from white to various shades of pink, pale purple, and red.

### Propagation of plant materials

The tops of 2–3 nodes of the plants were dipped in talc containing 1 g ·kg^− 1^ IBA (Indole-3-butyric acid, Sigma Chemical, Mo., U.S.A) for cutting propagation, and planted in BVB 7 H (7 H) mixed with commercial peat moss. PO441389, Bas van Buuren B V., Coldenhovelaan 10, The Netherlands) medium, transferred to 2-inch plastic pots containing the same medium after three weeks.

### Plant characteristics analysis

Photographs were taken when the test materials were in full bloom, focusing on the whole plant. The characteristics of the flowers and leaves were measured at the full-bloom flowers and the leaves in the middle of the plants respectively.

### DNA extraction

Leaves from the middle section of *Dianthus* were collected and ground with liquid nitrogen. CTAB (CetylTrimethyl Ammonium Bromide, USB corporation, U.S.A) was added for extraction, and the mixture was centrifuged at 23,180 ×g for 30 min. The supernatant as mixed with an equal amount of phenol: chloroform: isoamyl alcohol (25:24:1), thoroughly mixed, and centrifuged at 23,180 ×g for 15 min. The supernatant was then mixed with 75% alcohol, and the precipitated DNA pellet was dissolved in Tris-EDTA buffer (Lipp et al. 1999).

### Polymerase chain reaction (PCR)

The PCR reaction solution (25 µl) included 25 ng DNA template, 1x buffer, 2.5 mM dNTP, 0.2 µM primer, 1.25 units of Blend Taq, and H_2_O. For the ITS primer, refer to Zhang et al. (Table 2) (Zhang et al. 2002). The PCR reaction conditions were as follows: 94 °C for 10 min, 35 cycles of 94 °C for 30 s, 60 °C for 30 s, 72 °C for 90 s, and a final extension at 72 °C for 10 min. The RAPD PCR reaction used primers from Operon RAPD 10-mer Kit (Table 2). The PCR conditions were: 94 °C for 10 min, followed by 42 cycles of 94 °C for 45 s, 45 °C for 1 min, and 72 °C for 1 min, with a final extension at 72 °C for 10 min. PCR amplification products were analyzed using 1.5% agarose gel electrophoresis.

**Table 2:** Primers were used for ITS and RAPD phylogenetic analysis in 16 *Dianthus* spp

Primer	Sequence
**ITS**	
ITS1-ITS2	AGAAGTCGTAACAAGGTTTCCGTAGG
ITS2-ITS1	TACGGTGCGGATCAACCAGA
**RAPD**	
OPAC-10	TGTCTGGGTG
OPAI-01	GGCATCGGCT
OPAF-11	ACTGGGCCTC
OPAO-03	AGTCGGCCCA
OPAI-11	GGCACGCGTT
OPAB-04	ACATCGCCCA
OPP-08	TGAGAAGCGG
OPAV-08	ACCCGACCTG
OPAA-11	TCGCCCAGTC
OPA-20	GTGACGTAGG
OPQ-20	GACAGTCCCT
OPAD-09	TGGCCCTCAC
OPAE-19	GAAACGGGTG
OPK-12	GGGTAACGCC
OPM-07	CAGCACCCAC
OPA-07	TCTGTGCTGG
OPA-08	TCGCTTCTCC
OPA-09	AGGTGACCGT
OPA-13	GTTGCGATCC
OPA-14	CTCTCGGCGA
OPA-17	ACTGCGACCA
OPA-18	ACGGCGATGA
OPAG-14	CCGTGACTCA
OPAT-07	GACCGCTTGT
OPAX-01	GTGTGCCGTT

### Sequencing and phylogenetic analysis of the region of ITS

The ITS PCR products were sent to Genomics BioSci & Tech. Co.Ltd. for sequencing. The sequencing results were aligned using ClustalX software, and a dendrogram was constructed by performing 1000 bootstrap replications using the neighbor-joining method in MEGA4.

### RAPD genetic similarity and cluster analysis

The bands from the RAPD electrophoresis were analyzed using MVSP software (version 3.22). Clustering was performed using the UPGMA (unweighted pair-group method using arithmetic averages) method to construct a dendrogram.

## Data Availability

Not applicable.
